# Tazemetostat combined PD-1 inhibitor achieving prolonged survival in primary proximal epithelioid sarcoma: a case report and review of the literature

**DOI:** 10.1186/s13256-026-06312-4

**Published:** 2026-08-03

**Authors:** Wei Li, Chenlu Zhang, Yang You, Xi Guo, Rongkui Luo, Yuhong Zhou

**Affiliations:** 1 Department of Medical Oncology, Zhongshan Hospital, Fudan University, No. 180, Fenglin Road, Xuhui District, Shanghai, China; 2 Department of Pathology, Zhongshan Hospital, Fudan University, Shanghai, China

**Keywords:** Epithelioid sarcoma, Case report, Pelvic, INI-1, EZH2 inhibitor

## Abstract

**Background:**

Epithelioid sarcoma (ES) is a rare mesenchymal tumor categorized into proximal (axial) and classical (distal) types. In general, proximal ES come alone with limited treatment options and poor prognosis. Herein, we present the case of a proximal ES treated with a tazemetostat-containing regimen and achieved a favorable response in the third-line setting.

**Case description:**

A 44-year-old Han woman was noticed a left pelvic floor nodule by computed tomography (CT) scan, and histologically test result confirmed her diagnosis as epithelioid sarcoma (proximal type). Cell staining indicates a negative expression of INI1 but positive expression of PD-L1. Ifosfamide and adriamycin (IA) were introduced as first-line treatment because of multidisciplinary treatment (MDT) of soft-tissue tumor considered tumor invasion is extensive, not eligible to complete surgical resection. Unfortunately, IA was discontinued due to patient intolerance (severe myelosuppression). Anlotinib was used as second-line treatment after rapid disease progression and also discontinued due to vaginal hemorrhage. After the pathology diagnosis, the combination therapy of tislelizumab and tazemetostat was introduced. A significant tumor shrinkage was noticed after 21 months of follow-up, which achieved and continue to maintain a partial response (PR) with further tumor reduction.

**Conclusions:**

This case first reported a favorable response of primary proximal epithelioid sarcoma treated with EZH2 inhibitor tazemetostat and PD-1 inhibitor tislelizumab, suggesting a potential and well-tolerated treatment regimen for this rare disease.

## Introduction

Epithelioid sarcoma (ES) is a very rare subtype of soft-tissue tumors (STSs) first characterized by Enzinger in 1970, constituting approximately 1% of all sarcomas [1]. ES can be classified into two main types based on its anatomical location: classical and proximal. Classical ES typically affects the skin, subcutaneous tissue, or deeper soft tissues of the extremities in young adults. In contrast, proximal ES predominantly arises in axial locations and deep-seated lesions across a wide age range, often presenting with a poorer prognosis. These tumors are commonly found in proximal regions of the extremities, pelvis, perineum, and genital area [2]. Zegarra Buitron et al. analyzed 55 cases of proximal-type epithelioid sarcoma, with the pelvic region being the most frequently affected site (24 cases), followed by the adrenal gland and orbit (three cases each) [3].

ES is an aggressive tumor. The treatment for ES is very limited owing to the rarity of the tumor. Extensive surgical resection is still considered to be the most common method. In selected patients with localized disease, complete surgical resection can be potentially curative [4]. Patients with surgically unresectable local recurrence or metastatic disease are treated with traditional cytotoxic chemotherapy or tyrosine kinase inhibitors, with modest clinical benefit. Small, retrospective series have reported a median overall survival of 11–21 months in patients with metastatic disease [5–10]. Thus, novel, effective, and tolerable treatment strategies are needed. Here, we present a case of primary pelvic proximal-type ES treated with a combination of tislelizumab and tazemetostat, aiming to contribute additional therapeutic insights for the management of proximal-type ES.

## Case presentation

A 44-year-old Han woman with an Eastern Cooperative Oncology Group performance status (ECOG PS) score of 1 was admitted to our hospital in August 2022. The patient was initially considered with localized epithelioid low-grade spindle cell tumors and underwent systemic chemotherapy with IA at another hospital in May 2022. Unfortunately, chemotherapy was discontinued due to severe myelosuppression (grade 4 leukopenia, grade 4 neutropenia, grade 2 anemia) after one cycle. Subsequent CT imaging in August 2022 indicated disease progression. The patient then received anlotinib (12 mg orally once daily) until September 2022, discontinued due to vaginal hemorrhage.

**Fig. 1:**
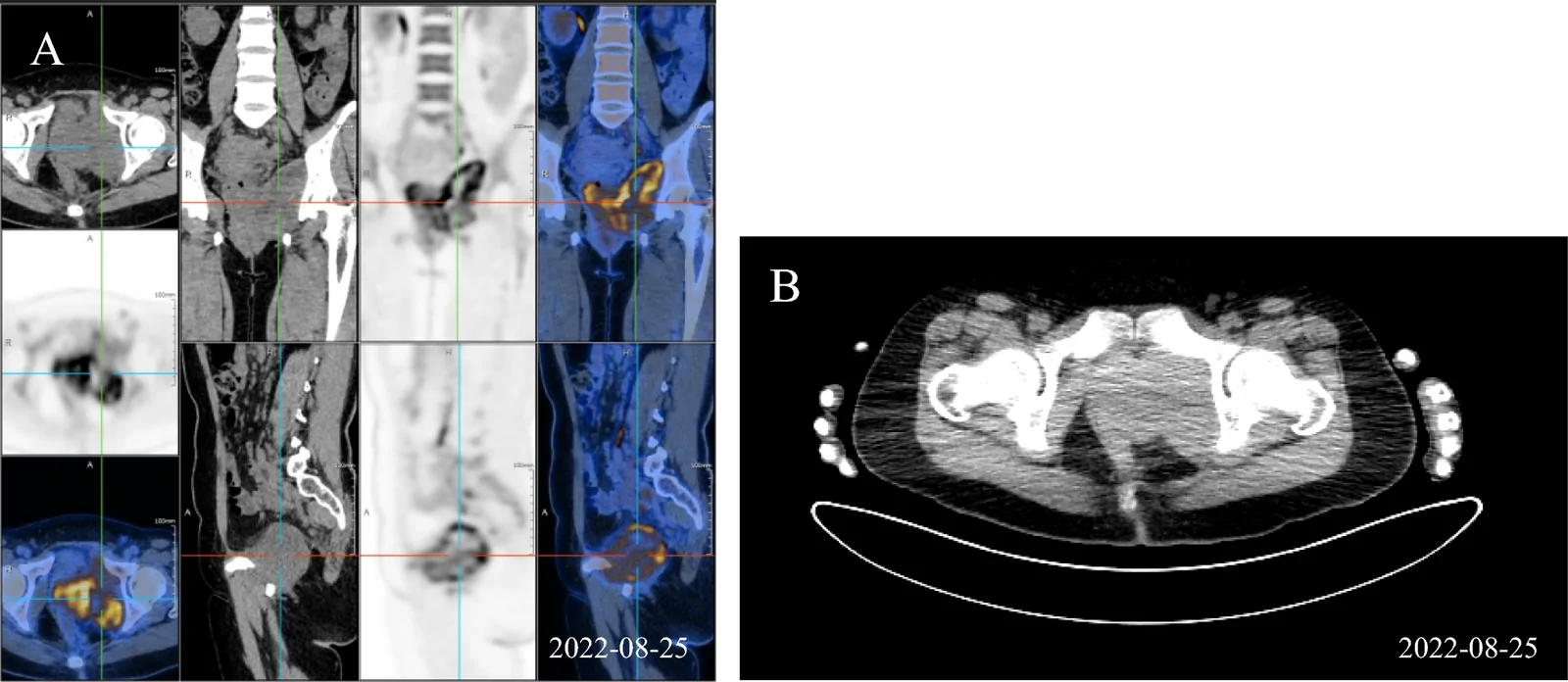
Imaging features of the left pelvic floor mesenchymal tumor. **A** PET/CT; **B** CT

**Fig. 2:**
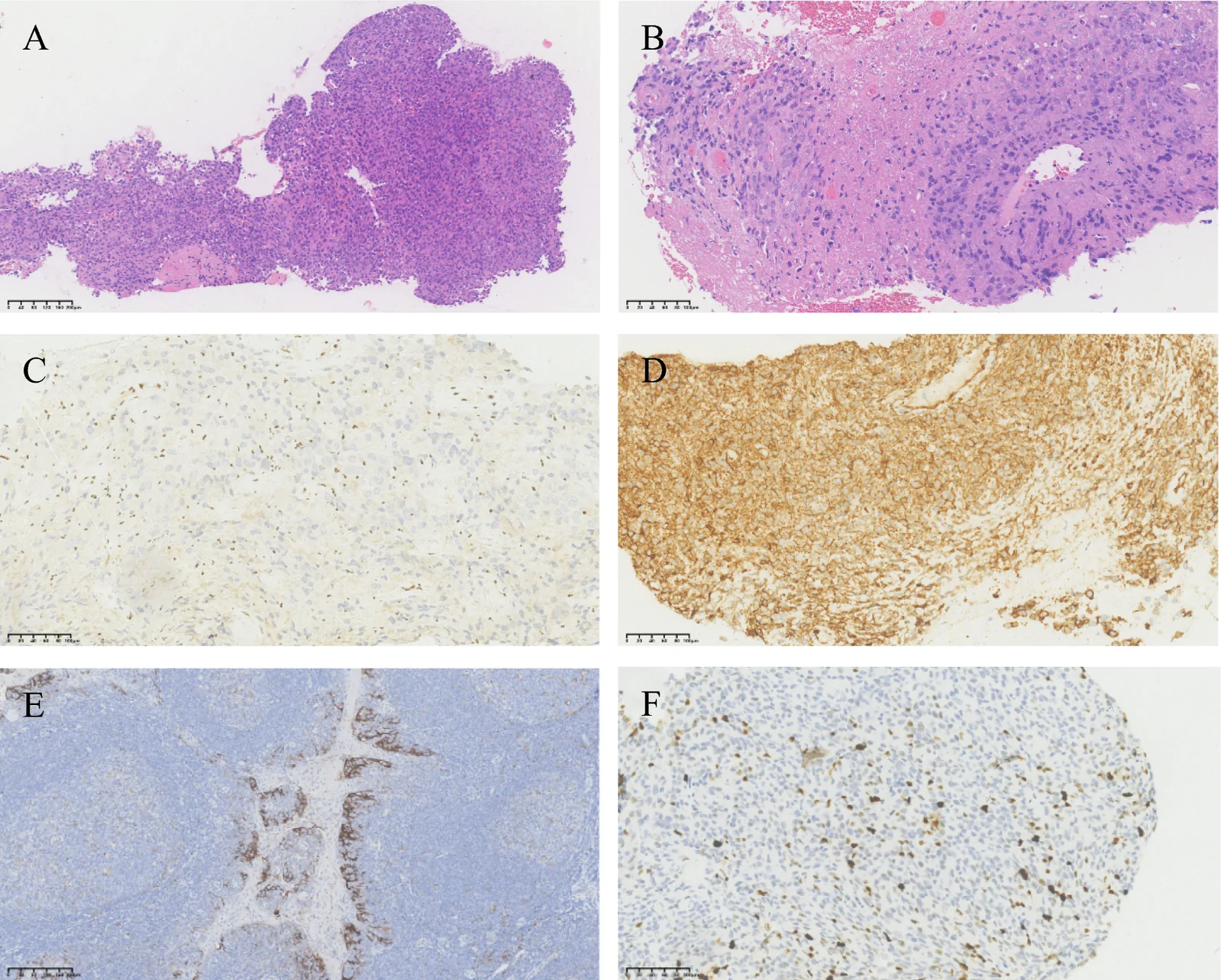
Hematoxylin–eosin (H&E) staining and immunohistochemical evaluation of the tumor tissue biopsy. **A**, **B** Hematoxylin–eosin staining, × 10 and × 20. **C**, **D**, **E**, **F** Immunohistochemical evaluation of INI1, CD34, PDL1, and Ki67

The patient underwent imaging evaluation and pathological consultation. As shown in Fig. 1, PET/CT demonstrated a hypermetabolic mesenchymal tumor in the left pelvic floor with invasion into the bladder wall, vaginal wall, left internal and external obturator muscles, and left pubic bone (Fig. 1A: PET/CT). Computed tomography (CT) confirmed a soft-tissue mass in the left pelvic floor (Fig. 1B:CT). Histopathologically, the tumor exhibited mesenchymal differentiation with pale vacuolated nuclei and mild cellular atypia. The mitotic count was approximately 3 per 10 high-power fields (HPFs), and focal necrosis was present. The tumor was classified as FNCLCC histological grade II (Fig. 2A, B; Hematoxylin–eosin staining, × 10 and × 20). Immunohistochemical (IHC) staining revealed the following results: positive for B- catenin(B-cat)( +), CD31( +), CD34( +), and BRG-1( +), while negative for Desmin(Des)(-), STAT6(-), S-100(-), smooth muscle actin (SMA)(-), ALK(1A4)(-), and pan-cytokeratin(CK)(-). In addition, the proliferative index Ki-67 was approximately 20%. Most importantly, loss of INI1 expression was observed. The tumor cells showed positive expression of the immune checkpoint pathway biomarker programmed death-ligand 1(PD-L1) (Fig. 2.C, D, E and F: Immunohistochemical evaluation of INI1, CD34, PDL1, and Ki67). The diagnosis of epithelioid sarcoma was eventually made, clinically staged as T2N1M0G2 IIIB.

**Fig. 3:**
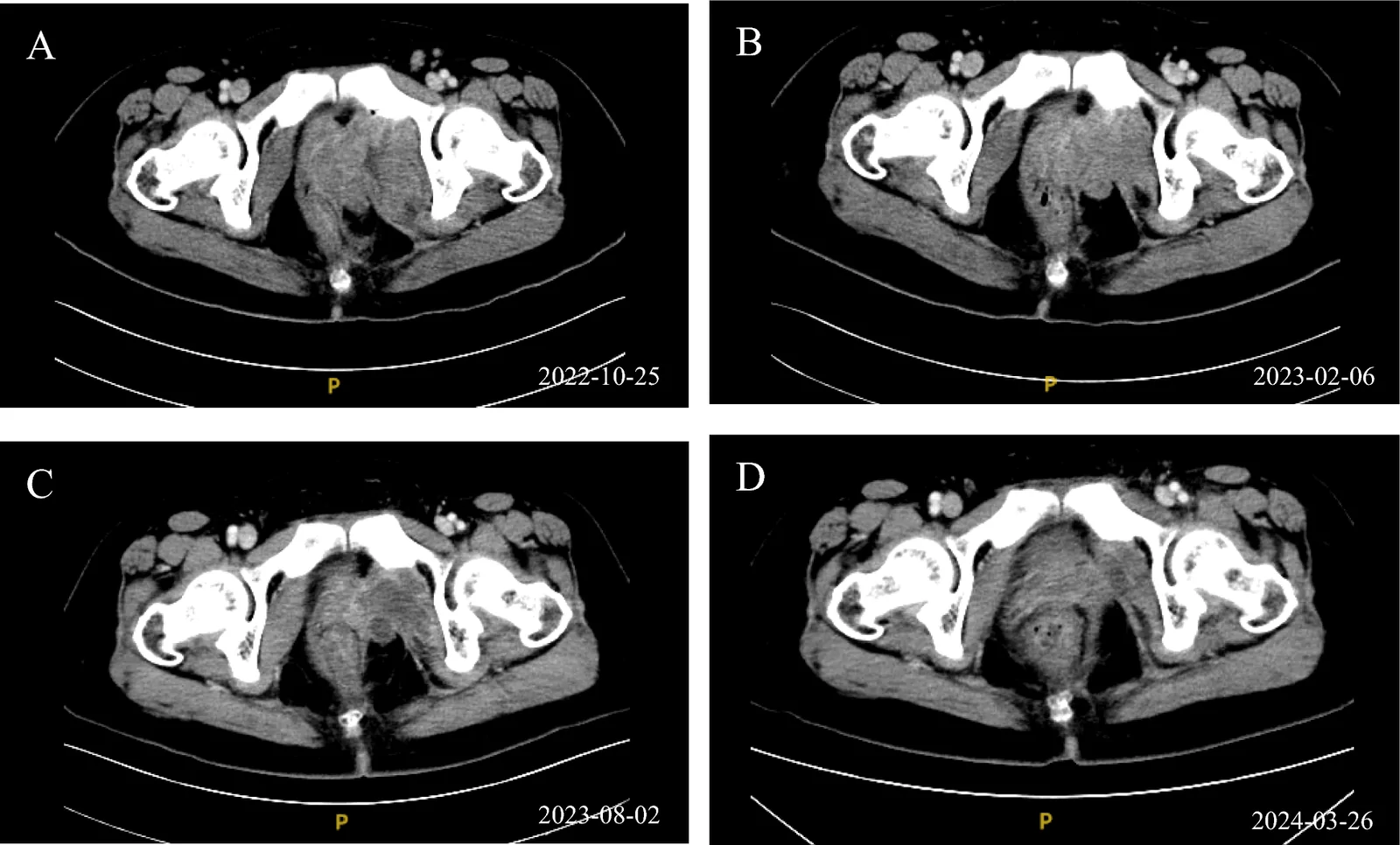
The sequential CT images illustrate the progressive tumor reduction during treatment. **A** October 2022; **B** February 2023; **C** August 2023; **D** March 2024

**Fig. 4:**
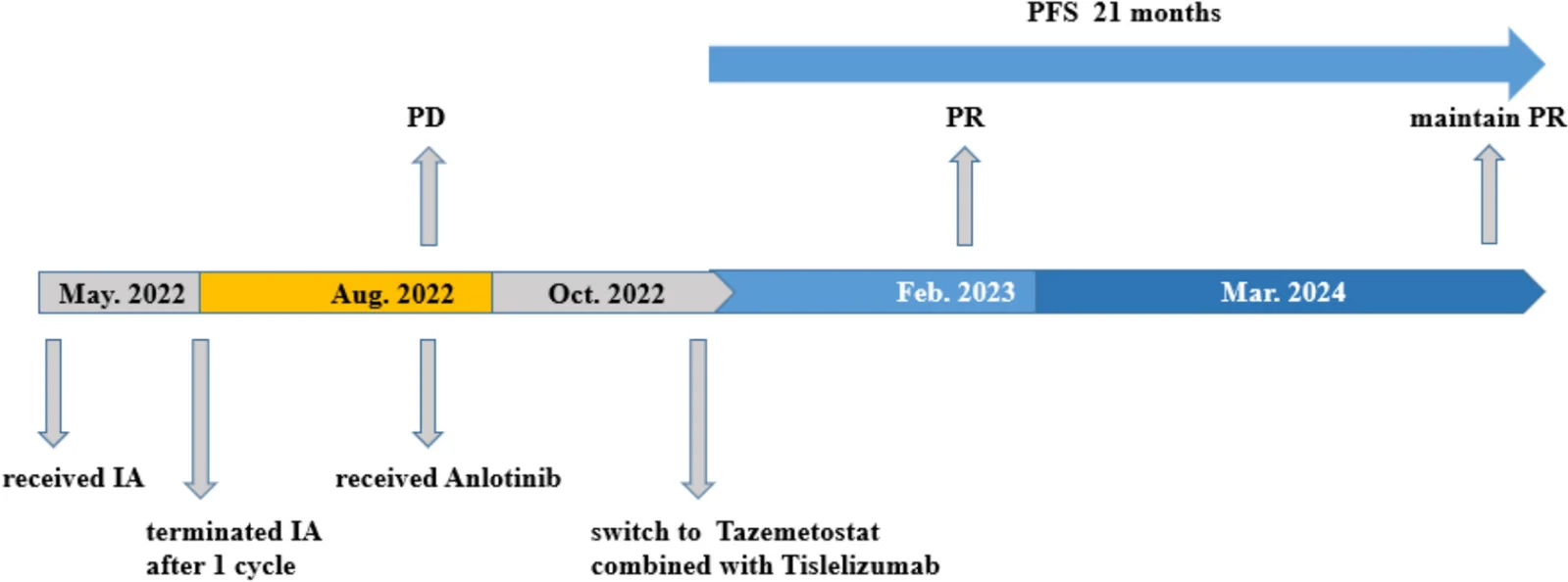
Treatment process and effect evaluation of the patient. *PD* progression disease, *PFS* progression-free survival, *PR* partial response

In October 2022, the patient was initiated on combination therapy consisting of tislelizumab (200 mg intravenously on day 1, every 3 weeks) and tazemetostat (800 mg orally twice daily). After 4 months of treatment, pelvic CT demonstrated marked tumor regression in the left pelvic floor, with the tumor size decreasing from 80 × 60 mm (October 2022) to 46 × 63 mm (February 2023), indicating a partial response (PR) per RECIST version 1.1 criteria. At the last follow-up in March 2024, CT showed further shrinkage to 44 × 31 mm (Fig. 3: The sequential CT images illustrate the progressive tumor reduction during treatment. (A) October 2022; (B) February 2023; (C) August 2023; (D) March 2024.). The primary treatment timeline is summarized (Fig. 4: treatment process and effect evaluation of the patient).

The patient experienced manageable treatment-related adverse events (TRAEs) including grade 2 hypothyroidism, grade 1 anemia, and grade 1 rash. No treatment discontinuation occurred due to AEs. Tislelizumab combined with tazemetostat demonstrated good tolerability. At the time of manuscript preparation, the patient continues to receive treatment with no evidence of disease progression. The combination therapy was well tolerated and resulted in an exceptional response in a patient with INI1-deficient epithelioid sarcoma.

## Discussion and conclusions

Epithelioid sarcoma is a rare malignancy, comprising approximately 1% of all sarcomas, and predominantly affects young to middle-aged adults (20–40 years) [11]. The proximal subtype is characterized by aggressive biological behavior, including higher recurrence rates, early metastasis, and increased mortality compared to the classical subtype. The 5-year overall survival of patients with primary, localized disease is approximately 60%, while the median survival of metastatic cases is 12–18 months [5, 12]. Complete surgical resection offers potential curative benefit for patients with localized disease [4]. However, for those with metastatic or locally advanced epithelioid sarcoma ineligible for complete resection, conventional cytotoxic chemotherapy yields limited responses [10]. Retrospective studies indicate a median overall survival of 11–21 months in patients with metastatic disease [8]. Jawad et al., analyzing 441 cases from the SEER Database, reported a 1-year survival rate of 46% [13].

Loss of INI1 (also known as SMARCB1, BAF47, and SNF5) occurs in more than 90% of ES cases, significantly enhancing diagnostic accuracy through immunohistochemistry [14, 15]. INI1, located at 22q11.23 and encoded by SMARCB1, is a critical core subunit of the SWI/SNF (Switch/ Sucrose Non-Fermentable) chromatin remodeling complex. This complex antagonizes the function of polycomb repressive complex 2 (PRC2), which plays a pivotal role in regulating cellular development through chromatin compaction and gene repression [16]. Loss of INI1 expression leads to aberrant activation of enhancer of zeste homolog 2 (EZH2). EZH2 is the catalytic subunit of PRC2, responsible for trimethylating lysine 27 of histone H3 (H3K27me3) [17]. EZH2 mediates the mono-, di-, and trimethylation of histone H3 lysine 27 (H3K27) by PRC2, with trimethylation repressing target gene transcription [18]. EZH2 or components of SWI/SNF complex aberrations can result in abnormal EZH2 expression or activity and subsequently an oncogenic dependence on EZH2 [19], while inhibiting PRC2 has been shown to decrease cell survival and tumor growth in various cancers [18]. EZH2 is considered the key oncogenic driver and has become a promising therapeutic target for ES [17].

Tazemetostat is small molecule enhancer of EZH2 inhibitor. In phase II study (NCT02601950, Registration Date: 2015–11-11), 62 patients with epithelioid sarcoma were enrolled in the study, patients received 800 mg tazemetostat orally twice per day in continuous 28- day cycles until disease progression, unacceptable toxicity, or withdrawal of consent. Nine (15%) of 62 patients had an objective response at the data cutoff. At a median follow-up of 13.8 months, median duration of response was not reached. 16 (26%) patients had disease control at 32 weeks. The median time to response was 3·9 months. The median progression-free survival was 5·5 months, and the median overall survival was 19·0 months. Grade 3 or worse treatment-related adverse events included anemia (four [6%]) and weight loss (two [3%]). Treatment-related serious adverse events occurred in two patients (one seizure and one hemoptysis). There were no treatment-related deaths [20]. Tazemetostat showed clinical activity in this cohort of patients with advanced epithelioid sarcoma characterized by loss of INI1/SMARCB1 and was approved in the USA for the treatment of patients with metastatic or locally advanced epithelioid sarcoma not eligible for complete resection, and was recommend as preferred regimens for epithelioid sarcoma systemic therapy agents by NCCN (National Comprehensive Cancer Network). Tazemetostat demonstrated good tolerability in patients, primarily causing grade 1–2 adverse effects and few dose reductions or treatment discontinuations. Notably, tazemetostat did not cause grade 3 or 4 nausea, thrombocytopenia, or neutropenia, which are common with traditional chemotherapy regimens [20]. These findings prompted the development of a phase 1b/3 randomized trial (NCT04204941, Registration Date: 2019–12-19), which aims to evaluate first-line therapy in epithelioid sarcoma patients randomized to receive doxorubicin combined with tazemetostat versus single-agent doxorubicin.

The role of immune checkpoint blockade in soft-tissue sarcomas is still under investigation, despite its transformative impact on the treatment landscape of many solid tumors. Kim et al. reported seven cases of ES with PD-L1 positivity (using a threshold of > 10% positivity in tumor cells), PD-L1 expression was linked to a shorter 5-year overall survival and an independent negative prognostic factor, that supports its role as a potential therapeutic target [21]. Tislelizumab, a humanized IgG4 monoclonal antibody targeting the PD-1 receptor, has demonstrated significant clinical benefit in a phase 2 trial involving patients with locally advanced or metastatic PD-L1 positive urothelial carcinoma [22]. Pembrolizumab and other immunotherapy agents have demonstrated moderate efficacy in patients with epithelioid sarcoma. In the KEYNOTE − 051 study, an 18- year-old patient with PD-L1-positive ES (PD-L1 40% positivity in the tumor cells) achieved a PR lasting approximately 12 months with pembrolizumab [23]. Similarly, a 24-year-old man with a proximal ES metastatic to the lung achieved PR after four cycles but progressed after eight cycles, another ES patient experienced disease progression at the first evaluation in a retrospective study of nivolumab [24].

In an effort to improving the efficacy of immune monotherapy for soft-tissue sarcomas, several combination strategies have been explored in clinical trials. Combined strategies aimed to modulate tumor microenvironment to enhance the effectiveness of PD-1/PD-L1 inhibitors in sarcomas. Prospective studies have primarily investigated combinations of PD-1/PD-L1 inhibitors with CTLA-4 checkpoint inhibitors, targeted therapies, as well as epigenetic or anti-angiogenic agents. We reviewed previous published data and ongoing trials of immune monotherapy and combination therapy for treatment of ES (Tables 1). Even though there are no head-to-head comparisons regarding PD-1/PD-L1 monotherapy and combination, it is commonly believed that PD-1/PD-L1 combination therapy always come up with a synergetic effect.

**Table 1:** Published data and ongoing trials evaluating immunotherapies monotherapy and combinations for ES

Intervention	Efficacy		References
	Sarcomas	ES	
Immunotherapy monotherapy			
Pembrolizumab	NA	1 ES: PR for 12 months	[23]
Nivolumab	24 STS: 1 PR	1 ES: PR for 4 months; 1 ES: PD	[24]
	38 advanced sarcomas: ORR 5%, mPFS 1.7 months	1 ES: PD	[30]
Immunotherapy combination with another immunotherapy			
Nivolumab + ipilimumab	38 advanced sarcomas: ORR 16%, mPFS 4.1 months	NA	[30]
Nivolumab + ipilimumab	NA	1 ES: CR	[29]
Immunotherapy combination with anti-angiogenic agents			
Pembrolizumab + axitinib	30 STS: ORR 25%, mPFS 4.7 months	1 ES: PR for 6 months	[31]
Nivolumab + sunitinib	58 STS: ORR 21%, mPFS 5.6 months	7 ES: No ORR, 1 SD	[32]
Durvalumab + pazopanib	46 STS: ORR 30.4%, mPFS 7.7 months	NA	[33]
TQB2450 + anlotinib	30 STS: ORR 36.7%, mPFS 7.8 months	1 ES: PR At the data cut-off still under treatment 14 months	[34]
Immunotherapy combination EZH2 inhibitor			
Durvalumab + tazemetostat	Phase II, cohort D: soft-tissue sarcoma	NA	NCT04705818 (Registration Date: 2021–01-12)
Pembrolizumab + tazemetostat	Phase I/II, advanced urothelial carcinoma	NA	NCT03854474 (Registration Date: 2019–02-26)
Nivolumab + Ipilimumab(aCTLA-4) + tazemetostat	Phase I/II, INI1- or SMARCA4-negative cancers	NA	NCT05407441 (Registration Date: 2022–06-07)

*STS* soft-tissue sarcoma, *ES* epithelioid sarcoma, *ORR* Objective response rate, *mPFS* median progression-free survival, *PR* partial response, *CR* complete response, *PD* progressive disease, *NA* Not Applicable

Preclinical studies have demonstrated that EZH2 inhibitors possess the potential to regulate tumor immunogenicity and anti-tumor immune responses, with their mechanisms of action closely linked to the modulation of immune escape phenotypes. Specifically, EZH2 facilitates tumor immune escape by activating the STAT3 pathway and suppressing the expression of CXCL9 and CXCL10, thereby inhibiting the activation of CD8 + T cells [25]. Furthermore, accumulating evidence has confirmed that EZH2 inhibitors can overcome primary and secondary resistance to PD-L1 inhibitors across multiple cancer types via these mechanisms [26, 27]. Consequently, tazemetostat is being extensively investigated as a potential immunomodulator.

The AcSe Pembro trial investigated pembrolizumab in ultra-rare sarcomas, reporting a complete response in one patient with epithelioid sarcoma and prolonged stable disease in three other ES patients. Furthermore, in a cohort of SMARCA4-deficient sarcomas or malignant rhabdoid tumors (MRTs), 3 out of 12 patients exhibited prolonged partial responses, suggesting increased sensitivity of SWI/SNF-defective sarcomas to anti-PD-1 antibodies [28]. Pecora A et al. reported a case of stage IV SMARCB1 inactivated ES with recurrent end stage (ECOG PS 4) rapidly progressing bulky disease previously treated with EZH2 inhibitors, achieving an unexpected complete response (CR) lasting over 11 months with nivolumab and ipilimumab therapy[29], Whether prior exposure to EZH2 inhibitors influences such responses warrants further investigation.

In our case, based on INI1 loss and positive PD-L1 expression, the patient underwent treatment with tislelizumab combined with tazemetostat. After four cycles of this combination therapy, CT imaging showed reduction of the primary lesion and was evaluated as a PR, indicating effectiveness of the combination treatment.

Our case provides an insight that the combination therapy of EZH2 and PD-1/PD-L1 inhibitor may have a synergetic effect and result in a significant survival benefit. However, this phenomenon needs to be explained and verified by clinical studies. An ongoing phase 2 CAIRE clinical trial (NCT04705818, Registration Date: 2021–01-12) is exploring the combination of the anti-PD-L1 agent durvalumab with tazemetostat in various solid tumors, including soft-tissue sarcoma, which may provide additional insights into the efficacy and safety of combining immunotherapy with EZH2 inhibitors in this context. These ongoing trials will deepen our understanding of the immunogenicity of ES and provide further evidence regarding the outcomes of immunotherapy combinations in this rare sarcoma subtype, aiding in the identification of patients who may benefit from specific immunotherapy regimens. A summary of current clinical trials exploring tazemetostat and immune therapies for ES is provided in Table 1.

In summary, epithelioid sarcoma represents a rare subtype of soft-tissue sarcoma characterized by clinical and behavioral heterogeneity. Managing advanced ES remains challenging due to its limited responsiveness to conventional chemotherapy regimens. The chemotherapy regimens which are similar to those commonly used in soft-tissue sarcomas as the standard first-line treatment is anthracycline-based chemotherapy [35]. In our case, the patient experienced severe myelosuppression and could not continue with IA chemotherapy. Given the patient’s loss of INI1 expression and positive PD-L1 status, we opted for treatment with tislelizumab combined with tazemetostat. Following 4 months of therapy, pelvic CT demonstrated a significant reduction in tumor size. Subsequently, over a 21-month follow-up period, the patient has sustained a PR with further improvement in response depth. Previous studies have reported a median progression-free survival (PFS) of 5.5 months and a 1-year PFS rate of 21% with single-agent tazemetostat [20], whereas the present case demonstrates the potential for long-term clinical benefit with combination therapy. Unfortunately, tazemetostat was withdrawn from the market in March 2026 due to the occurrence of secondary hematologic malignancies as adverse events observed in the SYMPHONY-1 phase Ib/III trial. Nevertheless, the clinical value of EZH2 inhibitors has been clearly evidenced in practice, warranting the development of safer EZH2-targeting agents for future clinical application.

## Data Availability

Data is provided within the manuscript.

## Abbreviations

ES: Epithelioid sarcoma
STS: Soft-tissue tumors
CT: Computed tomography
PET/CT: Positron emission tomography/computed tomography
IHC: Immunohistochemical
IA: Ifosfamide and adriamycin
MDT: Multidisciplinary treatment
CR: Complete response
ORR: Objective response rate
PR: Partial response
PD: Progression disease
PFS: Progression-free survival
mPFS: Median progression-free survival
PRC2: Polycomb repressive complex 2
EZH2: Enhancer of zeste homolog 2
TRAEs: Treatment-related adverse events
NA: Not applicable
